# Impaired Myocardial Function Is Prognostic for Severe Respiratory Failure in the Course of COVID-19 Infection

**DOI:** 10.3389/fcvm.2021.584108

**Published:** 2021-06-04

**Authors:** Alvaro Petersen-Uribe, Alban Avdiu, Peter Martus, Katja Witzel, Philippa Jaeger, Monika Zdanyte, David Heinzmann, Elli Tavlaki, Verena Warm, Tobias Geisler, Karin Müller, Meinrad Gawaz, Dominik Rath

**Affiliations:** ^1^Department of Cardiology and Angiology, University Hospital Tübingen, Eberhard Karls Universität Tübingen, Tübingen, Germany; ^2^Institute for Clinical Epidemiology and Applied Biostatistics, University Hospital Tübingen, Eberhard Karls Universität Tübingen, Tübingen, Germany

**Keywords:** COVID-19, mechanical ventilation, mortality, myocardial function, prognosis

## Abstract

COVID-19 may lead to severe acute respiratory distress syndrome (ARDS) resulting in increased morbidity and mortality. Heart failure and/or pre-existing cardiovascular disease may correlate with poor outcomes and thus require special attention from treating physicians. The present study sought to investigate a possible impact of impaired myocardial function as well as myocardial distress markers on mortality or ARDS with need for mechanical ventilation in 157 consecutive patients with confirmed SARS-CoV-2 infection. All patients were admitted and treated at the University Hospital of Tübingen, Germany, during the first wave of the pandemic. Electrocardiography, echocardiography, and routine blood sampling were performed at hospital admission. Impaired left-ventricular and right-ventricular function, tricuspid regurgitation > grade 1, and elevated RV-pressure as well as thrombotic and myocardial distress markers (D-dimers, NT-pro-BNP, and troponin-I) were associated with mechanical ventilation and/or all-cause mortality. Impaired cardiac function is more frequent amidst ARDS, leading to subsequent need for mechanical ventilation, and thus denotes a poor outcome in COVID-19. Since a causal treatment for SARS-CoV-2 infection is still lacking, guideline-compliant cardiovascular evaluation and treatment remains the best approach to improve outcomes in COVID-19 patients with cardiovascular comorbidities.

## Introduction

Severe acute respiratory syndrome coronavirus 2 (SARS-CoV-2) is an emerging cause of acute respiratory distress syndrome (ARDS) (1). Depending on the severity of ARDS, mechanical ventilation is the cornerstone for treatment of these critically ill patients (2). Patients in need for mechanical ventilation endure prolonged intra-hospital stay, neurological dysfunctions associated with concomitant anesthesia, and increased incidence of thrombosis and thromboembolism due to pro-thrombotic effects of SARS-CoV-2 and immobilization (3). Most importantly, severe respiratory failure is strongly associated with increased mortality in COVID-19 patients (4).

COVID-19 may cause severe acute myocardial injury or exacerbate an underlying chronic cardiovascular disease. Elevated levels of myocardial distress markers NT-pro-BNP and troponin are common findings in these patients (5). Moreover, pre-existing cardiovascular disease and compromised myocardial function have been associated with worse outcomes (6). Electrocardiography (ECG), echocardiography, and blood sampling for specific myocardial distress markers, e.g., troponin I and NT-pro-BNP, are essential for identifying COVID-19 patients with cardiovascular risk in order to improve management and consequently course of the disease. Since we currently lack a specific treatment for COVID-19, management of pre-existing or developing cardiac impairment is critical for improving outcomes in severely affected patients. Effects of impaired myocardial function on development of progressive respiratory failure and subsequent need for mechanical ventilation are unknown so far. Here, we report that markers of myocardial distress and impaired myocardial function are associated with progressive respiratory failure and increased mortality.

## Materials and Methods

### Study Design and Participants

In March and April 2020, this prospective study enrolled 157 consecutive patients diagnosed with severe COVID-19-associated respiratory failure, including the first wave of COVID-19 infections at the University Hospital of Tübingen, Germany. The aim of the current study was to enroll all COVID-19-positive patients requiring hospital admission. Hence, a confirmed SARS-CoV-2 infection requiring hospital admission represented the only selection criterion. According to our official hospital database, 187 patients with confirmed SARS-CoV-2 infection were treated in our university hospital in March and April 2020. We managed to include 84.0% of these COVID-19 patients into the current study. Within 24 h after hospital admission, an extensive cardiovascular assessment including ECG, transthoracic echocardiography (TTE), and testing for myocardial distress biomarkers (e.g., pro-NT-BNP and troponin I) was performed. Written informed consent was obtained wherever possible (*n* = 128, 81.5%). We strongly assume that the remaining patients would not have refused to participate in the study since the cardiologic assessment was performed routinely and not purely study associated. In mechanically ventilated patients, the patient consent was obtained once invasive ventilation was discontinued or after discharge. In these patients, no study-associated measurements were performed but already existing clinical data was analyzed in accordance with the local ethics committee. The study was approved by the institutional ethics committee (238/2018BO2) and complies with the Declaration of Helsinki and good clinical practice guidelines (7–9).

### Diagnosis of SARS-CoV-2 Infection and ARDS

SARS-CoV-2 was detected from nasopharyngeal secretions using real-time reverse transcriptase polymerase chain reaction. Severe respiratory failure was defined according to the Berlin Definition of Acute Respiratory Distress Syndrome (10).

### Twelve-Channel ECG and Laboratory Parameters

Twelve-channel ECG was registered according to standard procedure. Peripheral venous blood was drawn for routine laboratory parameters.

### Transthoracic Echocardiography

TTE was performed by our Cardio-COVID-19 team. Left ventricular ejection fraction (LVEF) was assessed visually and measured using Simpson's method (11). Impaired LVEF was defined as an EF <50% (12). Impaired right ventricular function (RV-function) was evaluated combining visual assessment and measuring of tricuspid annular plane systolic excursion (TAPSE). TAPSE was assessed by placing an M-mode cursor through the lateral tricuspid valve annulus in the apical four-chamber view. Then, the total systolic excursion distance of the tricuspid annulus was measured. Impaired RV-function was defined by TAPSE <20 mm (13). Mitral regurgitation was assessed based on regurgitant orifice area and width of vena contracta (14). Severity of aortic stenosis was defined based on valve area measured by continuity equation and planimetry (15). Jet/left ventricular outflow tract width ratio, pressure half time, as well as diastolic flow reversal in proximal descending aorta were used to quantify severity of aortic regurgitation (16). Central jet area and width of vena contracta were applied to determine tricuspid regurgitation (14). Right ventricular pressure was estimated using the simplified Bernoulli equitation [RVPsys = 4 × (Vmax)^2^] (17) when tricuspid regurgitation was present (18). High probability of pulmonary hypertension was defined as RV-pressure > 35 mmHg (19).

Finally, the presence of pericardial effusion (PE) was visually assessed (20).

### Clinical Follow-Up

All patients were followed up for 30 days after study inclusion for the primary combined endpoint (poor outcome): mechanical ventilation and/or mortality. Secondary endpoint included all-cause mortality and mechanical ventilation.

### Statistical Analysis

SPSS version 26.0 (SPSS Inc., Chicago, IL) and GraphPad Prism8.4.0 (GraphPad Software, San Diego, CA) were used for all statistical analyses. Student's *t*-test was applied for normally distributed data, whereas Mann–Whitney *U*-test served for analysis of non-normally distributed data. Accordingly, mean values are presented as mean ± standard deviation and median values are presented as median and 25th/75th percentiles. Categorical endpoints were analyzed *via* cross-tabulations and Chi-square tests. Correlations of non-normally distributed were assessed using Spearman's rank correlation coefficient (rho). Kaplan–Meier curves with log rank tests were applied to compare survival between groups, whereas multiple Cox regression analyses were used to analyse independent associations between myocardial distress markers and the combined endpoint after adjustment for epidemiological factors. Regarding Cox regression analyses, LVEF, RV-pressure, and age were included as continuous variables, whereas RV-function (normal vs. impaired), significant TR, arterial hypertension, coronary artery disease, as well as diabetes mellitus (no vs. yes) were coded as binary variables. Discriminatory performance of myocardial distress markers and other clinical factors was evaluated using receiver operator curves (ROC) and expressed as c-statistics with 95% CI. Depending on the area under the curve (AUC), ROC 0.5 suggests no discrimination, ≥0.7– <0.8 acceptable, ≥0.8– <0.9 excellent, and ≥0.9 outstanding discrimination (21). ROC analyses using combinations of predictors were based on multiple logistic regression analysis with leaving one out correction. Ninety-five percent CIs of these areas are included in the figures. Course of biomarkers and respective associations with poor outcome were analyzed *via* linear mixed-models with random intercept.

## Results

A total of 157 patients were included, and their baseline characteristics are shown in Table 1. Stratification according to incidence of the combined endpoint is presented in Table 2. Routine blood sampling was performed in the whole collective; ECG and echocardiography were performed in 136 (86.6%) and 133 (84.7%) patients, respectively. Rate of mechanical ventilation within 30 days after hospital admission was 44.6% (*n* = 70). Twenty (12.7%) patients developed severe ARDS in the course of the hospital stay. Twenty-two (14.0%) patients were already mechanically ventilated at admission. Twenty-eight (17.8%) patients were intubated due to rapidly increasing respiratory failure and for airway protection. A total of 25 patients died (15.9%); two patients died without being mechanically ventilated (1.3%).

**Table 1:** Baseline characteristics of the overall cohort (*n* = 157).

	**All**
	**(*n* = 157)**
Age, years (mean ± SD)	68 (±15)
Male, *n* (%)	99 (63.1)
Body mass index (mean ± SD)	29 (±5)
**Cardiovascular risk factors**, ***n*****(%)**	
Arterial hypertension	110 (70.1)
Dyslipidemia	55 (36.2)
Diabetes mellitus	36 (23.1)
Current smokers	7 (4.6)
Obesity	39 (25.8)
Atrial fibrillation	36 (23.1)
Known CAD	34 (22.4)
Chronic kidney disease	20 (12.7)
**Echocardiography**	
Left ventricular function, % (mean ± SD)	57 (±7)
Left ventricular hypertrophy, *n* (%)	94 (69.1)
Visually estimated normal right ventricular function, *n* (%)	112 (82.4)
Visually estimated impaired right ventricular function, *n* (%)	17 (12.5)
Right ventricular dilatation, *n* (%)	51 (37.5)
TAPSE, mm (mean ± SD)	22 (±5)
RV pressure, mmHg (mean ± SD)	29 (±11)
Aortic stenosis >1, *n* (%)	5 (3.7)
Aortic regurgitation >1, *n* (%)	12 (8.8)
Mitral regurgitation >1, *n* (%)	31 (22.8)
Tricuspid regurgitation >1, *n* (%)	34 (25.0)
Pericardial effusion, *n* (%)	64 (47.1)
**Electrocardiography**	
Rate, bpm (mean ± SD)	84 (±22)
Sinus rhythm, *n* (%)	108 (81.2)
QRS, ms (mean ± SD)	93 (±20)
Regular R progression, *n* (%)	78 (58.6)
Right bundle branch block, *n* (%)	4 (3.0)
Left bundle branch block, *n* (%)	2 (1.5)
PQ segment, ms (mean ± SD)	170 (±87)
QTc, ms (mean ± SD)	437 (±65)
Negative T wave, *n* (%)	14 (10.5)
ST segment depression, *n* (%)	2 (1.5)
ST segment elevation, *n* (%)	0 (0.0)
**Admission laboratory, median (25th percentile−75th percentile)**	
Leucocytes, 1,000/μL	6.6 (4.8–9.5)
Lymphocytes, 1,000/μL	0.8 (0.6–1.1)
Creatinine, mg/dL	0.9 (0.7–1.3)
GFR, mL/m^2^	74 (48–92)
D-Dimer, μg/dL	1.3 (0.7–2.8)
C-reactive protein, mg/dL	8.2 (2.6–16.0)
Procalcitonin, ng/mL	0.14 (0.07–0.74)
Troponin I, ng/dL	17 (6–56)
NT pro-BNP, ng/L	458 (139–2827)
CK, U/L	149 (74–346)
AST, U/L	43 (27–70)
ALT, U/L	32 (21–47)
LDH, U/L	337 (232–446)
**Medication at admission**, ***n*****(%)**	
Oral anticoagulation	21 (14.8)
ACEi/ARB	78 (54.9)
Aldosterone inhibitors	17 (12.0)
Diuretics	52 (36.6)
Calcium channel blockers	32 (22.5)
Beta blockers	58 (40.8)
Statins	51 (35.9)
ASA	36 (25.4)
P2Y12 blockers	3 (2.1)

**Table 2:** Baseline characteristics stratified according to the combined endpoint.

	**Combined endpoint**		
	**No (*n* = 85)**	**Yes (*n* = 72)**	***p* value**
Age, years (mean ± SD)	67 (±14)	68 (±16)	0.575
Male, *n* (%)	48 (56.5)	51 (70.8)	0.063
Body mass index (mean ± SD)	29 (±6)	29 (±5)	0.709
**Cardiovascular risk factors**, ***n*** **(%)**			
Arterial hypertension	53 (62.4)	57 (79.2)	**0.022**
Dyslipidemia	34 (40.0)	21 (29.2)	0.179
Diabetes mellitus	19 (22.4)	17 (23.6)	0.546
Current smokers	5 (5.9)	2 (2.8)	0.368
Obesity	21 (24.7)	18 (25.0)	0.870
Atrial fibrillation	15 (17.6)	21 (29.2)	0.095
Known CAD	14 (16.5)	20 (27.8)	0.311
Chronic kidney disease	9 (10.6)	11 (15.3)	0.380
**Echocardiography**			
Left ventricular function, % (mean ± SD)	59 (±4)	54 (±10)	**0.002**
Left ventricular hypertrophy, *n* (%)	57 (78.1)	37 (62.7)	0.514
Visually estimated normal right ventricular function, *n* (%)	71 (93.4)	41 (69.5)	**0.008**
Visually estimated impaired right ventricular function, *n* (%)	5 (6.6)	12 (20.3)	**0.008**
Right ventricular dilatation, *n* (%)	29 (39.7)	22 (37.3)	0.164
TAPSE, mm (mean ± SD)	22 (±5)	21 (±6)	0.441
RV pressure, mmHg (mean ± SD)	27 (±9)	32 (±12)	**0.045**
Aortic stenosis >1, *n* (%)	2 (2.7)	3 (5.1)	0.478
Aortic regurgitation >1, *n* (%)	7 (9.6)	5 (8.5)	0.989
Mitral regurgitation >1, *n* (%)	15 (20.5)	16 (27.1)	0.185
Tricuspid regurgitation >1, *n* (%)	13 (17.8)	21 (35.6)	**0.004**
Pericardial effusion, *n* (%)	32 (43.8)	30 (50.8)	0.180
**Electrocardiography**			
Rate, bpm (mean ± SD)	80 (±18)	88 (±26)	**0.029**
Sinus rhythm, *n* (%)	64 (84.2)	44 (77.2)	0.566
QRS, ms (mean ± SD)	93 (±23)	93 (±16)	0.931
Regular R progression, *n* (%)	47 (61.8)	31 (54.4)	0.385
Right bundle branch block, *n* (%)	2 (2.6)	2 (3.5)	0.877
Left bundle branch block, *n* (%)	2 (2.6)	0 (0.0)	0.243
PQ segment, ms (mean ± SD)	167 (±83)	173 (±93)	0.722
QTc, ms (mean ± SD)	427 (±81)	451 (±31)	**0.041**
Negative T wave, *n* (%)	4 (5.3)	10 (17.5)	**0.036**
ST segment depression, *n* (%)	1 (1.3)	1 (1.7)	0.821
ST segment elevation, *n* (%)	0 (0.0)	0 (0.0)	0.557
**Admission laboratory, median (25th percentile−75th percentile)**			
Leucocytes, 1,000/μL	5.7 (4.2–7.5)	7.7 (5-9–11.9)	** <0.001**
Lymphocytes, 1,000/μL	0.9 (0.7–1.2)	0.7 (0.5–1.0)	**0.005**
Creatinine, mg/dL	0.9 (0.7–1.2)	1.0 (0.8–1.6)	**0.027**
GFR, mL/m^2^	79.0 (58.9–97.2)	68.3 (37.5–91.6)	0.071
D-Dimer, μg/dL	0.8 (0.5–1.5)	2.4 (1.2–5.9)	** <0.001**
C-reactive protein, mg/dL	3.5 (1.3–8.7)	16.3 (9.2–27.4)	** <0.001**
Procalcitonin, ng/mL	0.08 (0.05–0.17)	0.58 (0.13–2.01)	** <0.001**
Troponin I, ng/dL	9 (4–18)	33 (18–124)	** <0.001**
NT pro-BNP, ng/L	310 (93–839)	1815 (401–6026)	** <0.001**
CK, U/L	121 (67–240)	273 (91–727)	**0.001**
AST, U/L	34 (20–47)	61 (39–102)	** <0.001**
ALT, U/L	28 (19–38)	41 (26–66)	** <0.001**
LDH, U/L	265 (207–361)	429 (337–494)	** <0.001**
**Medication at admission**, ***n*** **(%)**			
Oral anticoagulation	12 (14.1)	9 (12.5)	0.919
ACEi/ARB	44 (51.8)	34 (47.7)	0.640
Aldosterone inhibitors	9 (10.6)	8 (11.1)	0.642
Diuretics	29 (34.1)	23 (31.9)	0.701
Calcium channel blockers	19 (22.4)	13 (18.1)	0.843
Beta blockers	31 (36.5)	27 (37.6)	0.343
Statins	29 (34.1)	22 (370.6)	0.815
ASA	21 (24.7)	15 (20.8)	0.973
P2Y12 blockers	1 (1.2)	2 (2.8)	0.370

*Bold values indicate statistical significance*.

**Figure 1:**
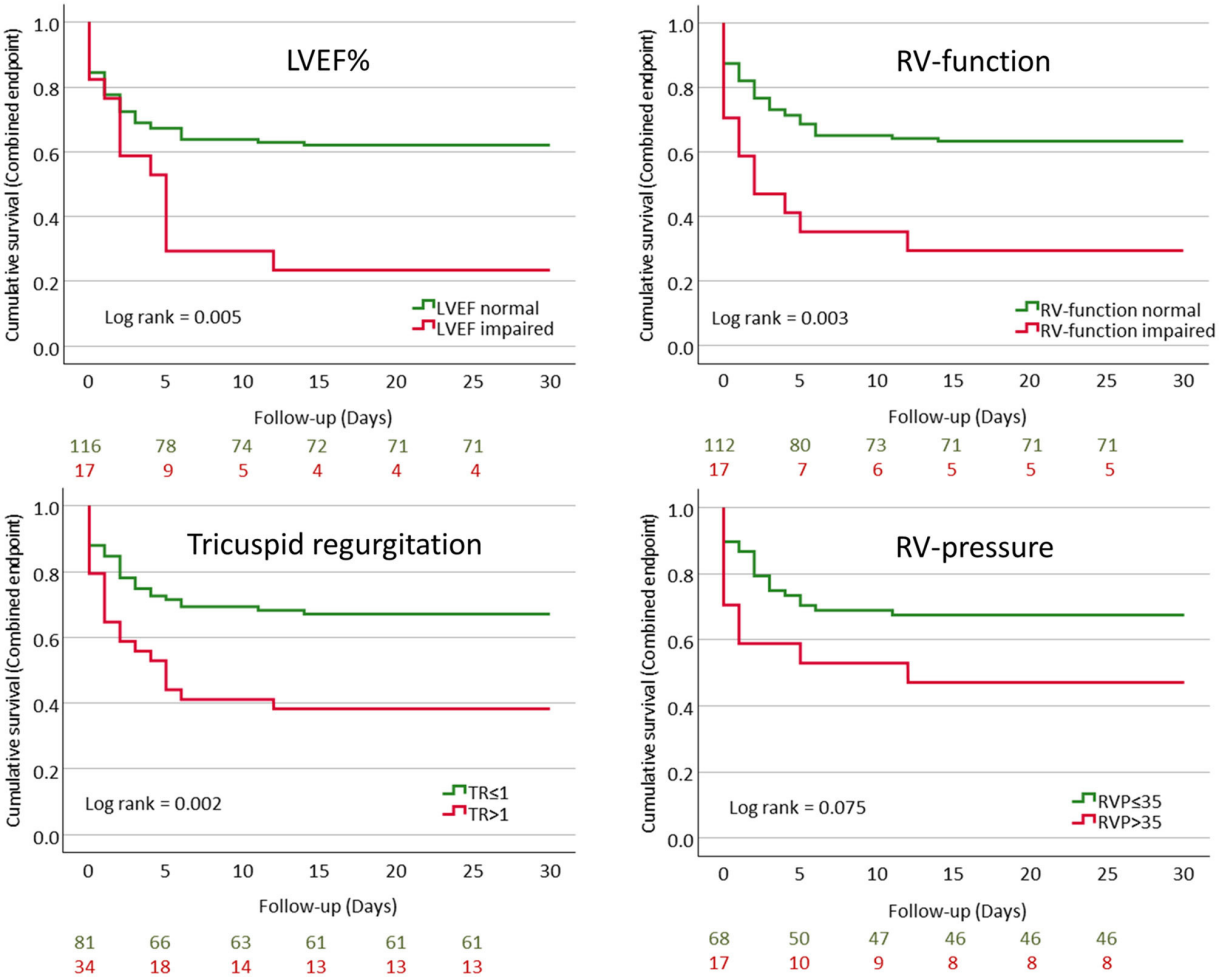
Kaplan–Meier curves showing cumulative event-free survival for the combined endpoint (mechanical ventilation and/or mortality) stratified according to LVEF%, RV-function, tricuspid regurgitation, and RV-pressure.

Patients with poor outcome displayed a significantly lower LVEF, worse RV-function, more severe tricuspid regurgitation, and increased RV pressure when compared to those with milder course of COVID-19 (Table 2 and Figure 1).

Multivariable Cox-regression analysis revealed that impaired LVEF and RV-function as well as tricuspid regurgitation >1 and increased RV-pressure were independently associated with poor outcome (Table 3).

**Table 3:** Cox regression with markers of myocardial function as well as epidemiological factors as independent variables and the combined endpoint as dependent variables.

	***p-*value**	**HR**	**95% CI**
Age	0.167	0.985	(0.964–1.006)
Arterial hypertension	0.321	1.456	(0.693–3.061)
Coronary artery disease	0.873	1.047	(0.594–1.845)
Diabetes mellitus	0.409	1.283	(0.709–2.321)
LVEF	**0.002**	0.955	(0.926–0.984)
Age	0.332	0.989	(0.969–1.011)
Arterial hypertension	0.292	1.496	(0.707–3.166)
Coronary artery disease	0.798	1.086	(0.579–2.037)
Diabetes mellitus	0.901	1.040	(0.563–1.922)
RV-Function	**0.010**	2.463	(1.239–4.895)
Age	0.062	0.977	(0.954–1.001)
Arterial hypertension	0.357	1.463	(0.651–3.288)
Coronary artery disease	0.335	1.312	(0.756–2.276)
Diabetes mellitus	0.505	1.233	(0.666–2.283)
Significant TR	**0.002**	2.851	(1.480–5.490)
Age	0.070	0.970	(0.939–1.002)
Arterial hypertension	0.074	3.085	(0.898–10.596)
Coronary artery disease	0.426	1.348	(0.647–2.808)
Diabetes mellitus	0.979	0.979	(0.451–2.170)
RV-pressure	**0.025**	1.040	(1.005–1.076)

*Bold values indicate statistical significance*.

Amidst patients with poor outcome, leucocyte count, D-dimers, C-reactive protein, procalcitonin, troponin I, NT-pro-BNP, CK, AST, and LDH levels were significantly higher when compared to COVID-19 patients with a more favorable course of disease (Table 2).

Increased QTc interval and a higher heart rate, just as a larger proportion of T wave inversion, were more frequently observed in patients requiring ventilation in the course of disease (Table 2). The locations of inverted T waves were distributed as follows: Lead I: *n* = 6 (42.9%), lead II: *n* = 2 (14.3%), lead III: *n* = 5 (35.7%), lead aVR: *n* = 10 (71.4%), lead aVL: *n* = 6 (42.9%), lead aVF: *n* = 5 (35.7%), lead V_1_: *n* = 6 (42.9%), lead V_2_: *n* = 5 (35.7%), lead V_3_: *n* = 7 (50.0%), lead V_4_: *n* = 6 (42.9%), lead V_5_: *n* = 4 (28.6%), and lead V_6_: *n* = 3 (21.4%), respectively.

**Figure 2:**
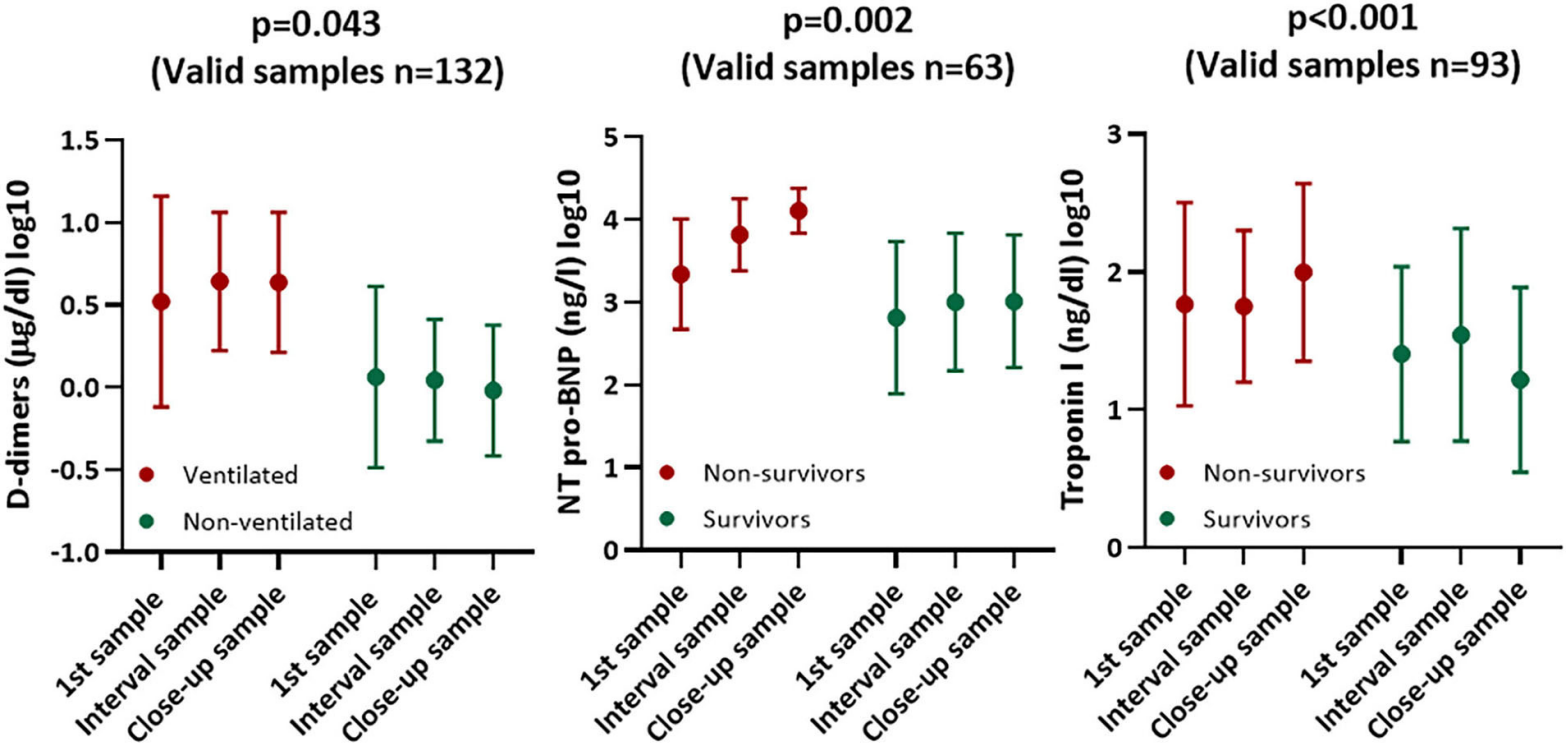
Diagrams (mean ± SD) showing course of cardiac and thrombotic biomarkers stratified according to survival and mechanical ventilation.

Mechanically ventilated patients showed significantly progressive D-dimer levels when compared to the remaining subjects (*p* = 0.043). Furthermore, non-survivors showed significantly progressive NT-pro-BNP and troponin-I levels when compared to survivors (*p* = 0.002 and *p* <0.001, respectively) (Table 4, Figure 2).

**Table 4:** D-dimer, troponin-I, and NT-pro-BNP levels at admission (1st sample), median of hospital stay (interval sample), and discharge/death (close-up sample) stratified according to mechanical ventilation, all-cause mortality, and the combined endpoint.

		**1st sample**	**Interval sample**	**Close-up sample**	***p-*value** **(int)**	***p-*value** **(time)**	***p-*value** **(group)**
**Mechanical ventilation**							
D-dimers (±SD)	Ventilated	10.3 (±18.1)0.5 (±0.6)	7.3 (±9.0) 0.6 (±0.4)	7.1 (±8.8)0.6 (±0.4)	**0.043**	0.760	** <0.001**
	Non-ventilated	4.1 (±11.6)0.1 (±0.5)	1.7 (±2.6) 0.0 (±0.4)	1.7 (±2.9)−0.2 (±0.4)			
Troponin-I (±SD)	Ventilated	167 (±412)1.7 (±0.6)	351 (±1061) 1.8 (±0.7)	202 (±586)1.6 (±0.7)	0.345	**0.023**	** <0.001**
	Non-ventilated	38 (±66)1.1 (±0.6)	40 (±73) 1.2 (±0.6)	32 (±48)1.1 (±0.6)			
NT pro-BNP (±SD)	Ventilated	6894 (±9695)3.4 (±0.8)	9114 (±8691) 3.6 (±0.8)	11623 (±12862)3.7 (±0.8)	0.985	** <0.001**	** <0.001**
	Non-ventilated	7818 (±39032)2.6 (±0.9)	7352 (±36658) 2.8 (±0.8)	6550 (±29070)2.9 (±0.7)			
**All-cause mortality**						
D-dimers (±SD)	Non-survivors	9.6 (±14.3)0.5 (±0.7)	5.6 (±5.4) 0.6 (±0.3)	7.4 (±7.1)0.7 (±0.4)	0.258	0.360	** <0.001**
	Survivors	6.7 (±15.7)0.2 (±0.6)	4.5 (±7.7) 0.3 (±0.5)	3.8 (±6.9)0.2 (±0.5)			
Troponin-I (±SD)	Non-survivors	244 (±535)1.8 (±0.7)	141 (±253) 1.8 (±0.5)	352 (±816)2.0 (±0.6)	** <0.001**	0.860	**0.002**
	Survivors	84 (±248)1.4 (±0.6)	264 (±949) 1.5 (±0.8)	82 (±311) 1.2 (±0.7)			
NT pro-BNP (±SD)	Non-survivors	5064 (±6750)3.3 (±0.7)	8931 (±5697) 3.8 (±0.4)	16023 (±15442)4.1 (±0.3)	**0.002**	** <0.001**	**0.002**
	Survivors	7854 (±34447)2.8 (±0.9)	7819 (±31940) 3.0 (±0.9)	7137 (±25882)3.0 (±0.8)			
**Combined endpoint (CE)**						
D-dimers (±SD)	CE yes	10.3 (±18.0)0.5 (±0.6)	7.2 (±9.0) 0.6 (±0.4)	7.1 (±8.7)0.6 (±0.4)	0.070	0.779	** <0.001**
	CE no	4.0 (±11.7)0.1 (±0.5)	1.7 (±2.6) 0.0 (±0.4)	1.7 (±3.0)−0.0 (±0.4)			
Troponin-I (±SD)	CE yes	167 (±412)1.7 (±0.6)	352 (±1062) 1.8 (±0.7)	202 (±586)1.6 (±0.7)	0.345	**0.023**	** <0.001**
	CE no	38 (±66)1.2 (±0.6)	40 (±73) 1.2 (±0.6)	32 (±48)1.1 (±0.6)			
NT pro-BNP (±SD)	CE yes	6894 (±9695)3.4 (±0.8)	9114 (±8691) 3.6 (±0.8)	11623 (±12862)3.7 (±0.8)	0.985	** <0.001**	** <0.001**
	CE no	7818 (±39032)2.6 (±0.9)	7352 (±36658) 2.8 (±0.8)	6550 (±29070)2.9 (±0.7)			

*Both raw and logarithmic values are shown. P-values were calculated for logarithmic data. Int, interaction. Bold values indicate statistical significance*.

**Figure 3:**
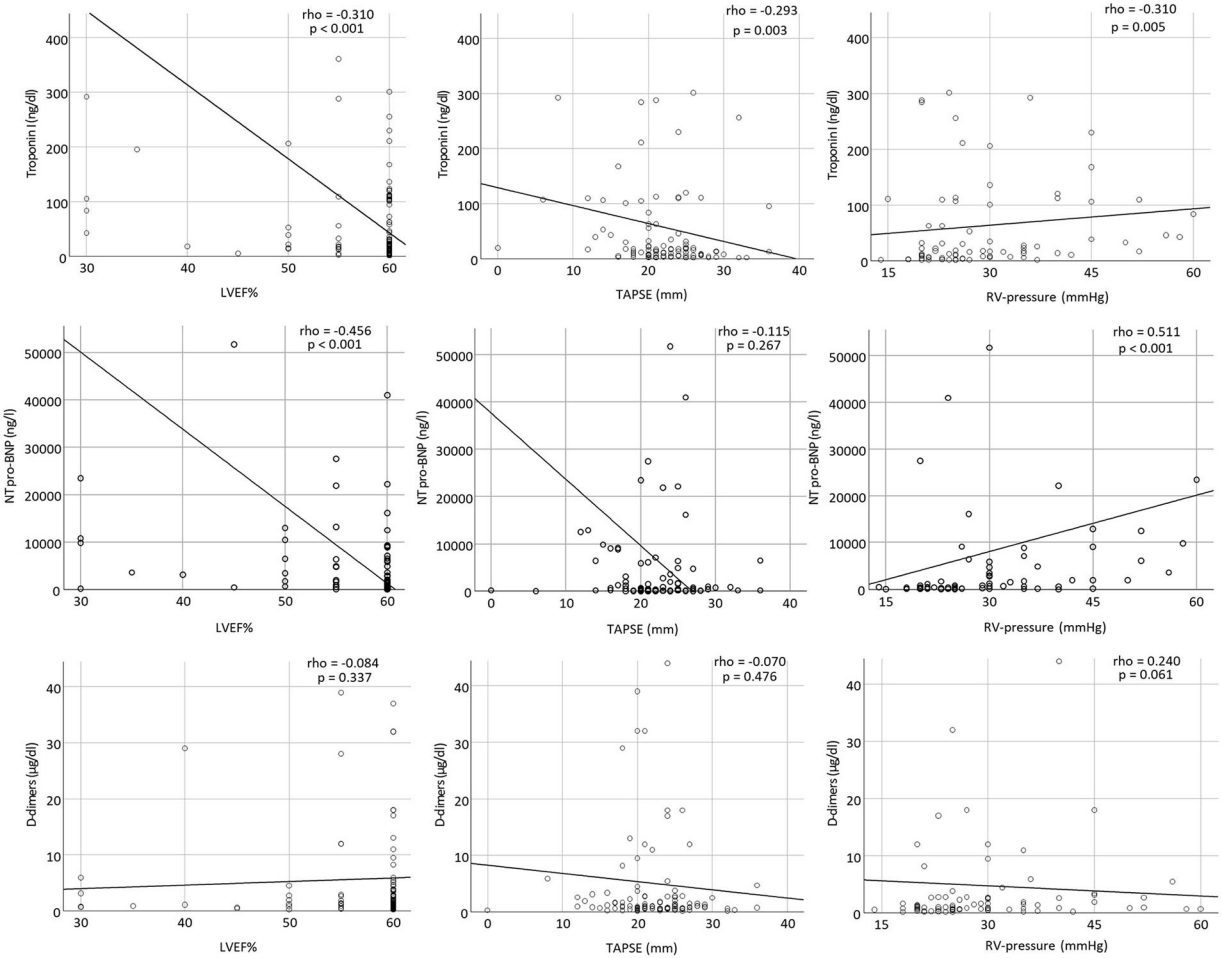
Scatter plots showing correlations between troponin I, NT-pro-BNP, and D-dimers with LVEF%, TAPSE, and RV-pressure at admission.

LVEF correlated significantly with troponin I and NT-pro-BNP at admission (rho = −0.310, *p* <0.001 and rho = −0.456, *p* <0.001, respectively). TAPSE correlated significantly with troponin I (rho = −0.293, *p* = 0.003). Finally, RV-pressure was significantly associated with troponin I and NT-pro-BNP (rho = 0.310, *p* = 0.005 and rho = 0.511, *p* <0.001, respectively) (Figure 3).

**Figure 4:**
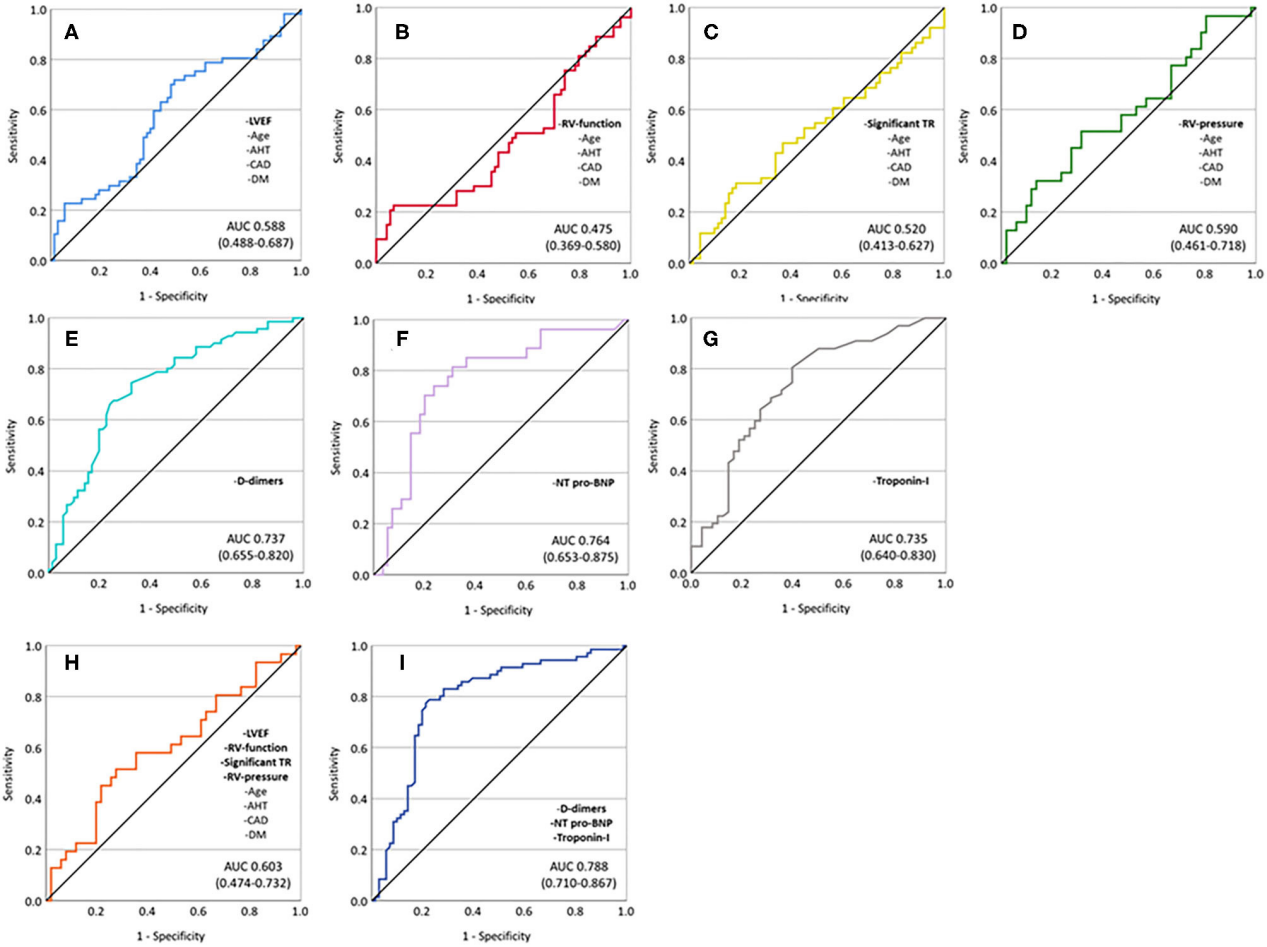
ROC analyses showing predictive performance of different univariate and multivariable models. **(A)** Age, arterial hypertension, coronary artery disease, diabetes mellitus, and impaired LVEF; **(B)** age, arterial hypertension, coronary artery disease, diabetes mellitus, and impaired RV-function; **(C)** age, arterial hypertension, coronary artery disease, diabetes mellitus, and significant TR; **(D)** age, arterial hypertension, coronary artery disease, diabetes mellitus, and elevated RV-pressure; **(E)** D-dimers; **(F)** NT-pro-BNP; **(G)** troponin-I; **(H)** age, arterial hypertension, coronary artery disease, diabetes mellitus, impaired LVEF, impaired RV-function, significant TR, and elevated RV-pressure; and **(I)** D-dimers, NT-pro-BNP, and troponin-I.

ROC analyses (combined endpoint) revealed an AUC of 0.588 for a multivariable model containing age, arterial hypertension, coronary artery disease, diabetes mellitus type II, and LVEF, 0.475 for a combination of age, arterial hypertension, coronary artery disease, diabetes mellitus type II, and RV-function, 0.520 for age, arterial hypertension, coronary artery disease, diabetes mellitus type II, and significant TR, and 0.590 for age, arterial hypertension, coronary artery disease, diabetes mellitus type II, and elevated RV-pressure. Cardiac biomarkers and D-dimer showed significantly better predictive performance (AUC 0.737 for D-dimers, 0.764 for NT-pro-BNP, and 0.735 for troponin-I). The best discrimination performance was achieved by a model including D-dimers, NT-pro-BNP, and troponin-I (AUC 0.788), whereas a combined model including age, arterial hypertension, coronary artery disease, diabetes mellitus type II, LVEF, RV-function, significant TR, and elevated RV-pressure performed poorly in predicting the combined endpoint (AUC 0.603) (Figure 4).

## Discussion

The major findings of this study are as follows: (1) Early impaired left and right ventricular systolic function, higher degree tricuspid regurgitation, and higher RV-pressure are more prevalent among COVID-19-positive patients with poor outcome. (2) The course of the myocardial distress markers NT-pro-BNP and troponin-I may predict outcome in COVID-19 patients. (3) Troponin I and NT-pro-BNP correlate with LVEF, RV-function, and RV pressure at admission. (4) A combined model including D-dimers, troponin-I, and NT-pro-BNP may facilitate risk assessment in COVID-19 patients.

The current findings provide further evidence that an extensive cardiologic assessment of patients suffering from COVID-19 is required at the earliest time point before severe respiratory symptoms are evident.

Our current data and previous reports emphasize that myocardial injury represents a prevalent finding in COVID-19 patients with respiratory insufficiency. Severe respiratory failure and ARDS are currently considered as the main cause of COVID-19-associated morbidity and mortality (22). Recently, Richardson and collaborators reported that 12.2% of hospitalized COVID-19 patients require mechanical ventilation (23). Among those, up to 20% developed cardiac injury, defined as an increase in troponin I (24). Interestingly, in our consecutive collective, ~45% of patients required mechanical ventilation, with 24% showing significant troponin I elevation. Susceptibility to SARS-CoV-2 infection seems to be higher in patients with pre-existing cardiovascular disease (25). Furthermore, these patients suffer from increased morbidity and mortality (26).

As the precise mechanisms leading to myocardial damage in COVID-19 await a thorough investigation, current research suggests that myocardial damage may result from direct viral or inflammatory myocardial injury and may be augmented by systemic inflammatory response, which further promotes microcirculatory impairment or arrest (22). Diagnosing COVID-19-induced direct myocardial damage is a challenging process requiring myocardial biopsy as a gold standard, although SARS-CoV-2 genome could not be identified within the myocardium in biopsy and autopsy findings so far (27). Furthermore, cardiac MRI may help identify myocarditis as a cause of impaired LV-function. These two diagnostic modalities were, however, not applied in our department during the first COVID-19 wave due to patient overload and protection of clinic personnel.

**Figure 5:**
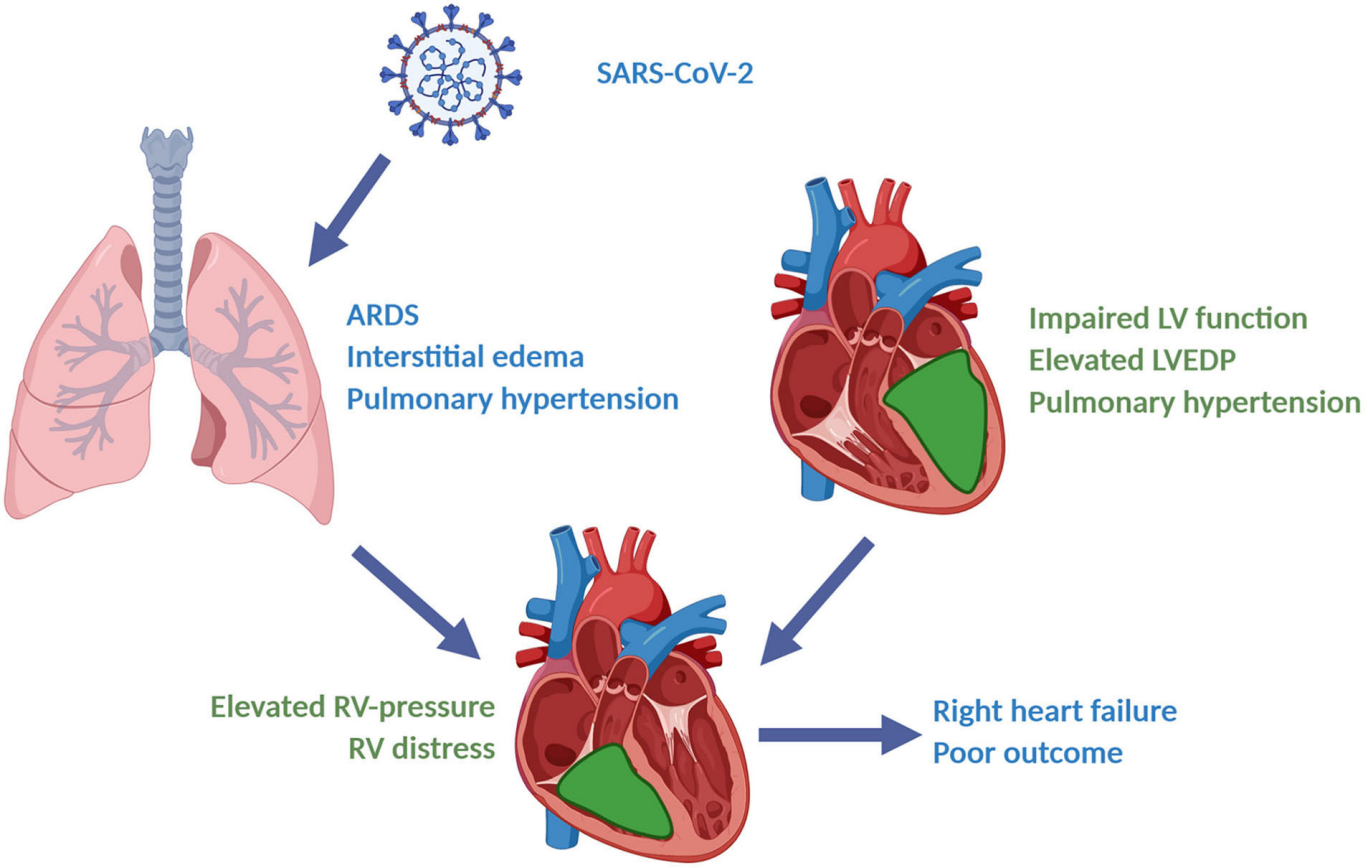
Right heart failure caused by COVID-19-induced severe ARDS and preexisting left heart failure: A hypothesis. (Figure created with BioRender®).

According to available echocardiography findings prior to the diagnosis of SARS-CoV-2 infection, impaired systolic LV-function was commonly a chronic condition, whereas impaired RV-function tended to be a new finding in the current patient cohort. This suggests that elevated RV-pressure and RV-dysfunction is an acute process caused by COVID-19-induced ARDS. Significantly elevated BNP and troponin-I levels found in mechanically ventilated COVID-19 patients in our cohort support the development of acute right ventricular failure, which is consistent with recent findings (28, 29). Furthermore, pulmonary distress could fittingly account for QRS prolongation and higher amount of abnormal T waves seen in our collective (30). Impaired LV-function at hospital admission may fasten this process by congestion caused by elevated LVEDP and thus raising pulmonary artery pressure. Our hypothesis of COVID-19-induced right ventricular failure as a result of major hemodynamic stress is presented in Figure 5.

## Conclusion

As cardiovascular comorbidities and myocardial injury significantly contribute to mortality in COVID-19, early cardiologic assessment and identification of high-risk patients is of critical importance to optimize the management and improve prognosis of COVID-19 patients.

### Limitations

The current study offers several major limitations. First, we could not differentiate between COVID-19-induced and non-COVID-19-induced impairment of myocardial function, which may have affected outcome to an unknown degree. Second, the number of patients enrolled was low, rendering generation of risk prediction models difficult. Third, we were not able to include all COVID-19-positive patients admitted to our hospital during the first wave of the disease. Finally, biomarker levels were not available for all patients.

## Data Availability Statement

The raw data supporting the conclusions of this article will be made available by the authors, without undue reservation.

## Ethics Statement

This study was approved by the institutional ethics committee (238/2018BO2) and complies with the declaration of Helsinki and good clinical practice guidelines. The patients/participants provided their written informed consent to participate in this study.

## Author's Note

We submitted this manuscript at the end of the first COVID-19 wave at the University Hospital Hospital of Tübingen. We have previously submitted an interimanalysis of COVID-19 positive patients to a different journal, which has been accepted for publication onMay 28th (Rath et al., Clin Res Cardiol 2020 Jun 14:1-9). The endpoint differed however and the number of events was significantly lower. Fewer patients were enrolled. Finally, additional analyses were performed in the current manuscript.

## Author Contributions

AP-U and AA: data collection, data analysis, and drafting of the manuscript. PM: expert data analysis. KW, PJ, MZ, DH, ET, VW, TG, and KM: data collection and critical revision. MG: study concept and drafting of the manuscript. DR: data collection, and data analysis, drafting of the manuscript, and study concept. All authors contributed to the article and approved the submitted version.

## Conflict of Interest

The authors declare that the research was conducted in the absence of any commercial or financial relationships that could be construed as a potential conflict of interest.
